# Colorimetric Detection of Class A Soybean Saponins by G-Quadruplex-Based Hybridization Chain Reaction

**DOI:** 10.1155/2020/8813239

**Published:** 2020-11-05

**Authors:** Congcong Yin, Qiaoling Zhao, Aiqin Yue, Weijun Du, Dingbin Liu, Jinzhong Zhao, Yongpo Zhang, Min Wang

**Affiliations:** ^1^College of Arts and Sciences, Shanxi Agricultural University, Taigu 030801, Shanxi, China; ^2^College of Agronomy, Shanxi Agricultural University, Taigu 030801, Shanxi, China; ^3^College of Chemistry, Nankai University, Tianjin 300071, China

## Abstract

Soybean saponin is one of the important secondary metabolites in seeds, which has various beneficial physiological functions to human health. *GmSg-1* gene is the key enzyme gene for synthesizing class A saponins. It is of great significance to realize the visual and rapid detection of class A saponins at the genetic level. The hybridization chain reaction (HCR) was employed to the visual detection of *GmSg-1* gene, which was implemented by changing the length of the target fragment to 92 bp and using the hairpin probes we designed to detect the *GmSg-1*^*a*^ and *GmSg-1*^*b*^ genes. The best condition of HCR reaction is hemin (1.2 *μ*M), Triton X-100 (0.002%), ABTS (3.8 *μ*M), and H_2_O_2_ (1.5 mM). It was found that HCR has high specificity for *GmSg-1* gene and could be applied to the visual detection of different soybean cultivars containing Aa type, Ab type, and Aa/Ab type saponins, which could provide technical reference and theoretical basis for molecular breeding of soybean and development of functional soybean products.

## 1. Introduction

Soybean not only contains high-quality nutrients such as protein, oil, vitamin, and dietary fiber but also contains bioactive substances such as soybean saponin, soybean isoflavone, soybean phospholipid, and anthocyanin, which are beneficial to human health. As the main secondary metabolites in soybean seed, soybean saponins have become a research hotspot. Medical research has proved that soybean has many physiological functions, such as significantly reducing blood lipid, antiatherosclerosis, antioxidation, and antitumor and could be developed into health foods or medicines [1, 2]. However, it is difficult to separate, purify, and detect saponins because of their various types and complex structures. At present, the direct detection methods of soybean saponin mainly include thin-layer chromography (TLC) [3], aglycone colorimetry [4], high-performance liquid chromatography (HPLC) [5, 6], high-speed counter-current chromatography (HSCCC) [7], and high-performance liquid chromatography-electrospray ionization-tandern mass spectrometry [8]. It is worth noting that each of these methods has its own shortcomings. For example, TLC can quickly screen and detect the content and composition of soybean saponins, but the results are not accurate enough; aglycone colorimetry cannot determine the content of total saponins; HPLC-MS needs corresponding standards, and the cost of operation is high. Therefore, it has become an urgent need to develop a simple and accurate method for qualitative and quantitative detection of soybean saponins. In recent years, the visual detection of DNA technology has attracted the attention of researchers because of its intuitive and rapid detection of genes [9]. Moreover, due to the advantages of simple, rapid, and sensitive and without the participation of natural enzymes, great efforts have been made in exploring the potential application of colorimetric assays in scientific research and clinical diagnosis, for example, detection of small-molecular antibiotic residues, quantitative screening of small-molecular mycotoxins, enzyme-free sensitive detection of alpha-fetoprotein (AFP), and so on [10–14].

As an important achievement in the development of modern molecular biology, genetic analysis has been widely used in the detection and identification of substances and could also be used as a reliable method for identification of class A saponins. In order to get more tested products in the shortest time to improve the sensitivity of detection, the majority of target genes are amplified by PCR; thus the detection of target genes could be transformed into the detection of PCR products for electrophoresis observation. However, this detection method is prone to false-positive results and requires complicated manipulations. In 2004, the hybridization chain reaction (HCR) proposed by Dirks and Pierce [15] is an enzyme-independent nucleic acid detection technology, which can realize the detection of nucleic acids at room temperature with only one pair of hairpin DNA probes. Compared with proteases, DNA probes are cheap, stable, and easy to store; compared with PCR and other amplification technologies, HCR could be completed at room temperature without strict reaction conditions and special equipment. These advantages are of great significance to simplify operation and make HCR system an ideal platform for developing nucleic and detection based on visual chip. Due to its advantages such as high-efficiency, isothermal amplification, ultra-high sensitivity, structural versatility, and enzyme-free properties, HCR has become a powerful molecular biology tool with wide application prospects in the fields of biosensing, bioimaging and biomedicine [16–18]. Thus far, HCR has been used for sensitive detection of various targets, including nucleic acids (DNA and RNA) [19], proteins [20, 21], enzyme activities [22], small biomolecules [23–25], metal ions [26, 27], and even tumor cells [28, 29]. Dong et al. [30] found that the gene polymorphism could be detected via the integration of HCR and G-quadruplex; the G-quadruplex released by HCR could combine with hemin chloride and color development, thus achieving target gene detection. This method has the advantages of high sensitivity, fast speed, and simplicity. However, it has not been reported to be applied to the identification of class A soybean saponins. *GmSg-1* gene is the key enzyme gene for synthesizing class A saponins. *GmSg-1* gene is subdivided into *GmSg-1*^*a*^ and *GmSg-1*^*b*^. *GmSg-1*^*a*^ gene encodes xylose transferase, which connects xylose at the C-22 terminal of saponin Aa, while *GmSg-1*^*b*^ gene encodes glucosyltransferase where glucose is conjugated to the C-22 terminal of saponin Ab. Studies have found that glycosylation occurs at C-22 and C-3 positions on daidzein. The key enzymes regulating the synthesis of saponins Aa and Ab, xylosyltransferase (UGT73F4), and glucosytransferase (UGT73F2) are encoded by two alleles of *GmSg-1*^*a*^ and *GmSg-1*^*b*^ [31]. In this study, we applied the integration of HCR and G-quadruplex to identify the two key enzyme genes. The purpose is to explore the feasibility of visual qualitative analysis of soybean saponins by HCR, which is of great significance for the identification of class A saponins in soybean germplasm resources, and provides a basis for molecular breeding of soybean and development of functional soybean products.

## 2. Experimental Section

### 2.1. Materials and Reagents

Hemin, ABTS, and H_2_O_2_ were purchased from Aladdin (Shanghai, China). Lambda exonuclease was purchased from Fermentas. Taq DNA polymerase, Taq buffer, and dNTP were purchased from Beijing TransGen Biotech Co., Ltd. (Beijing, China). All DNA sequences (Table 1, Supporting information) were synthesized and PAGE purified by Shanghai Sangon Biological Engineering Technology and Services (Shanghai, China). Tris, Triton X-100, NaCl, KCl, HCl, and NaOH were purchased from Heowns. Pipettes, microplate readers, and PCR amplifiers were purchased from Eppendorf. Canon camera was made by Canon. Gel imager was made by Shanghai Jiapeng Technology. Ball mill was made by Guobang mining machinery. All the solutions were prepared with sterile ultrapure water.

### 2.2. Instruments

PCR (Eppendorf AG), electrophoresis (Bio-RAD powerpac Basic), nuclear acid analyzer (Eppendorf Kinetic), gel imaging system (BIO-GENER GT9612), freeze dryer (Hetopowerdry), cryogenic refrigerator (Haier), and electronic balance (Shimadzu).

### 2.3. DNAzyme-Based PCR Amplification

The PCR was carried out in a mixture containing 4.2 *μ*L DNA template (20 ng/*μ*L), 3.0 *μ*L Taq buffer (10×), 0.6 *μ*L primer1 (TA92F, 10 *μ*M) and 0.6 *μ*L primer2 (TA92R, 10 *μ*M); 2.4 *μ*L dNTP (2.5 *μ*M), 0.6 *μ*L Taq DNA polymerase, and 0.6 *μ*L sterile water were added to make a final volume 30 *μ*L. The PCR procedure was as follows: 95°C for 5 min; 95°C 1 min, 6°C 45 s, 72°C for 8 s, 35 cycles; and 72°C for 10 min. The target sequence of 92 bp was obtained and detected by 3% agarose gel electrophoresis.

To obtain single-stranded target fragments required for HCR, an enzyme digestion reaction was performed: 1 *μ*L lambda exonuclease was added to 19 *μ*L PCR products, 37°C, 90 min and 85°C, 15 min. The digested products were detected by 3% agarose gel electrophoresis.

### 2.4. Colorimetric Detection

The hairpins probes need to be pretreated as follows: GA1 (1.5 *μ*L, 10 *μ*M) and GA2 (1.5 *μ*L, 10 *μ*M) were mixed with 5 × Tris/HCl-Na buffer (2.0 *μ*L, Tris 100 mM, NaCl 2 M, pH 7.2), respectively, and then heated to 95°C for 5 min and cooled down to ambient temperature for 1 h before use.

The total volume of HCR system was 10 *μ*L, containing enzyme digestion product (8 *μ*L), the pretreated probes GA1 (0.5 *μ*L) and GA2 (0.5 *μ*L), and 0.5 *μ*L HCl-NaCl (0.04 M HCl, 4 M NaCl) to maintain the pH of the reaction system. The reaction was performed for 1 h at 37°C and then terminated on ice. After that, KCl (0.135 *μ*M), hemin (1.2 *μ*M), and Triton X-100 (0.002%) were added into the reaction mixture, and then followed by the addition of ABTS (3.8 mM) and H_2_O_2_ (1.5 mM), the color changes were observed at about 10 min, and the results were detected by 3% agarose gel electrophoresis. By using this method, six soybean cultivars, Wuhei, Jinda 73, Jinyi 30, Jinke 4, WS285, and Jinda 47, were detected with two pairs of probes GAa1/GAa2 and GAb1/GAb2, respectively.

### 2.5. Visual-Chip-Based Assay of *GmSg-1*

The visual-chip carrier was made by a thick transparent plastic sheet which was washed thoroughly and sterilized with alcohol. Six gene chips were fixed in two rows on the plastic sheet, three in each row, each circle with a diameter of 0.8 cm. The pretreated probes GAa1/GAa2 and GAb1/GAb2 were mixed with 5 × Tris/HCl-Na buffer (Tris 100 mM, NaCl 2 M, pH 7.2), respectively; 3 *μ*L each of the solution was taken and mixed completely. Then the mixed solution was pipetted onto the chip and dried at 37°C to obtain the visual chip ready for target detection. Target fragment TAa (0.1 *μ*L, 10 *μ*M) and target fragment TAb (0.1 *μ*L, 10 *μ*M) were pipetted into the upper and lower circle on the visual chip, respectively. The blank circles were printed on sterile ultrapure water (0.1 *μ*L) instead of target fragment. The reaction was also carried out at 37°C for 1 h. After that, KCl (0.135 *μ*M), hemin (1.2 *μ*M), and Triton X-100 (0.002%) were pipetted onto the chip; two minutes later, ABTS (3.8 mM), H_2_O_2_ (1.5 mM) was added in the drops and color differences were observed.

## 3. Results and Discussion

### 3.1. The Principle of DNAzyme-Based HCR Assays for *GmSg-1* Genes

**Scheme 1:**
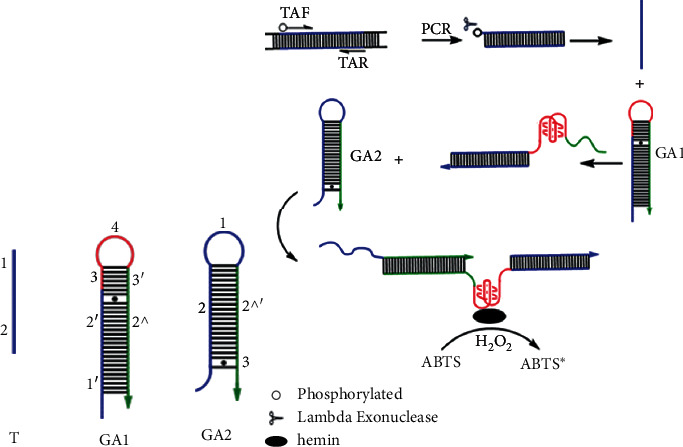
Schematic diagram of hybridization chain reaction. (a)Target fragment and structure of hairpin probes. The target should be single-stranded DNA. Sequences marked with the same number with or without a single quote (') are complementary to each other, and the punctuation (^) indicates that there is only one base difference between them.(b) The process of detecting *GmSg-1* by HCR reaction. In the absence of target, the structures of hairpin probes GA1 and GA2 are metastable and G-quadruplex is enclosed in the loop. Once the target is present, probes GA1 and GA2 are opened successively and hybridization chain reaction was generated, lots of G-quadruplex are released and combined with hemin to catalyze the oxidation of ABTS by H_2_O_2_ to green ABTS^+^.

The principle of *GmSg-1* gene detection based on HCR is illustrated in Scheme 1. To implement our strategy, we designed the sequences (TF, TR, 92 bp) of targeted GmSg-1a and *GmSg-1*^*b*^, and two pairs of complementary specific hairpin probes GAa1/GAa2 and GAb1/GAb2 according to the target recognition region (the sequences are shown in Table 1). Complementary sequences are marked by the same number with or without a single quote ('). The punctuation (^) indicates that there is only one base difference. In the hairpin probe GA1, the sequence of 1′-2′ is complementary to the 1–2 of the target sequence T, 3–4 is G-quadruplex fragment with two-thirds locked in the loop. In the hairpin probe GA2, the sequence of 1–2 is identical with the 1–2 of the target sequence T.

Firstly, the 5′-phosphorylated strand of duplex DNA was amplified by PCR before hybridization chain reaction; the PCR products were digested by Lambda Exonuclease that could specifically recognize and cleave the 5′-terminal phosphorylated DNA polymers; thus the double-stranded DNA could be turned into a single strand. When the target fragment is absent in the reaction system, the structures of pretreated probes GA1 and GA2 are metastable and the G-quadruplex is blocked in the hairpin structure and has no activity. However, when the target fragment is present, a cascade amplification reaction could be proceeded. Because 1′-2′ of probe GA1 is completely complementary to 1–2 of the target sequence T, the probe GA1 could be opened to release the G-quadruplex 3–4 and the sequence 3′-2^ that can trigger the probe GA2 to open. After the probe GA2 is opened, the same sequence 1–2 can play the same role as the target fragment. In this way, GA2 and GA1 are opened successively; the result is that the formation of long-stranded DNA polymeric molecules and DNA polymers bearing lots of G-quadruplex in between are produced. The G-quadruplex combined with hemin has catalase activity that can catalyze the oxidation of ABTS by H_2_O_2_, turning it into green ABTS^+^. The results could be observed directly or determined by spectrophotometer at 420 nm.

### 3.2. Validation of the HCR Assay for *GmSg-1* Genes

**Figure 1:**
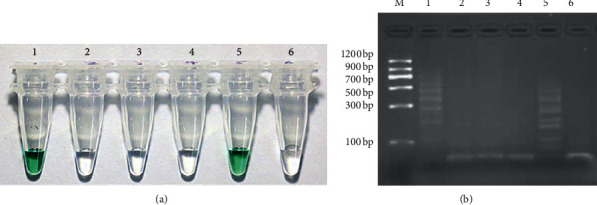
Colorimetric detection of the *GmSg-1*^*a*^ and *GmSg-1*^*b*^ using the HCR assays. (a) The photograph illustrates the colorimetric results of tubes 1–6 by adding different hairpin probes and target genes. Tube 1: GAa1 + GAa2 + TAa. Tube 2: GAa1 + GAa2 + TAb. Tube 3: blank. Tube 4: GAb1 + GAb2 + TAa. Tube 5: GAb1 + GAb2 + TAb. Tube 6: blank. (b) The corresponding results of 3% agarose gel electrophoresis.

To demonstrate the feasibility of the detection method for *GmSg-1* gene, three pairs of pretreated hairpin probes GAa1 (1.5 *µ*L, 10 *µ*M) and GAa2 (1.5 *µ*L, 10 *µ*M) were added into target sequence TAa (0.1 *µ*L, 10 *μ*M), TAb (0.1 *µ*L, 10 *μ*M), and sterile ultrapure water (0.1 *µ*L), respectively. The treatment group that replaced the target sequence with sterile ultrapure water served as the blank group. Water was added to HCR system to make a final volume of 20 *µ*L. The same treatment was done for hairpin probes GAb1 and GAb2. After the completion of hybridization chain reaction, color reaction was carried out as described in 2.4, and then the experimental results were recorded by taking photos and 3% agarose gel electrophoresis. The results showed that, in the presence of target gene TAa, the assay solution of HCR containing probes GAa1 and GAa2 turned green rapidly (Figure 1(a)) and the corresponding electrophoresis results (Figure 1(b)) showed that HCR occurred and yielded a set of long-chain polymers with different molecular weights. However, when probes GAb1 and GAb2 were present, the assay solution remained colorless under the same conditions; the corresponding electrophoresis results showed that only the remaining probes were found, and no polymers with different molecular were produced. In parallel, when the target gene was replaced by TAb, the similar results were achieved with high specificity. The results demonstrate that HCR not only has the feasibility of the colorimetric assay but also has a strong ability to differentiating gene sequence, so it could be used to identify *GmSg-1*^*a*^ and *GmSg-1*^*b*^ in different soybean cultivars.

### 3.3. Qualitative Detection of *GmSg-1* Genes from Soybean Cultivars

**Figure 2:**
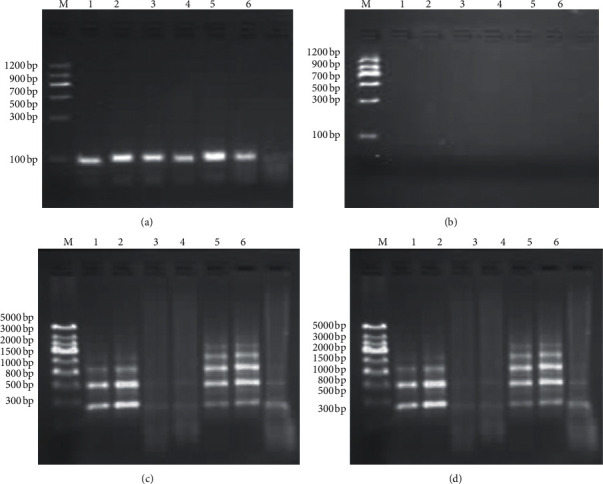
Electropherogram of target gene fragment in HCR. The lanes on both sides of the gel are DNA markers; lanes 1–6 represent six soybean cultivars in sequence: Wuhei, Jinda 73, Jinyi 30, Jinke 4, WS 285, and Jinda 47 in HCR system. (a) Double-strand target gene (TF/TR) amplified by PCR. (b) Agarose gel analysis of the PCR product digested by lambda exonuclease. (c) Agarose gel analysis of HCR products in the presence of probes GAa1 and GAa2. (d) Agarose gel analysis of HCR products in the presence of probes GAb1 and GAb2.

**Figure 3:**
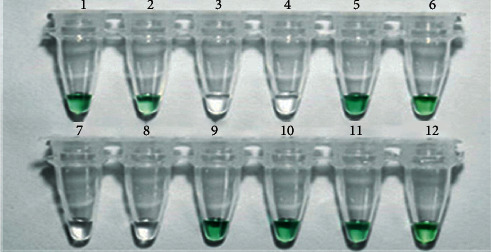
Colorimetric detection results for soybean *GmSg-1*^*a*^*/GmSg-1*^*b*^ genes. Photograph of HCR assay solutions containing hemin (1.2 *μ*M), Triton X-100 (0.002%), ABTS (3.8 *μ*M), H_2_O_2_ (1.5 mM), and target genes *GmSg-1*^*a*^ and *GmSg-1*^*b*^ collected from different soybean cultivars. Tubes 1, 7: Wuhei. Tubes 2, 8: Jinda 73. Tubes 3, 9: Jinyi 30. Tubes 4, 10: Jinke 4. Tubes 5, 11: WS 285. Tubes 6, 12: Jinda 47.

Based on the determination of soybean saponin content by HPLC-ESI-MS/MS, two soybean cultivars containing only Aa saponin (Wuhei and Jinda 73), two soybean cultivars containing only Ab saponin (Jinyi 30 and Jinke 4), and two soybean cultivars containing both Aa and Ab  WS 285 and Jinda 47) were selected to detect *GmSg-1*^*a*^ and *GmSg-1*^*b*^ genes by HCR. According to the *GmSg-1*^*a*^ and *GmSg-1*^*b*^ gene sequences, universal primers were designed to determine the target fragment. The target fragment length that could be amplified was finally selected to be 92 bp with the aid of NUPACK software. As seen from agarose gel analysis results (Figure 2(a)), stable double strands (TF/TR, 92 bp) were obtained by PCR amplification with the *GmSg-1*^*a*^ and *GmSg-1*^*b*^ sequences that were collected from the six cultivars mentioned above. With the aid of lambda exonuclease, the double strands were cleaved into single strands, which were also confirmed by the agarose gel analysis (Figure 2(b)). To perform the test, hemin (1.2 *μ*M), Triton X-100 (0.002%), ABTS (3.8 mM), and H_2_O_2_ (1.5 mM) were added to the mixtures of hybridization chain reaction containing varied soybean cultivars. In the presence of probes GAa1 and GAa2, the color of solution containing GmSg-1a genes turned into green in 10 min. In detail, tubes 1, 2, 5, and 6 containing Wuhei, Jinda 73, WS 285, and Jinda 47, respectively, showed obvious change from colorless to green (Figure 3). In contrast, tubes 3 and 4 containing Jinyi 30 and Jinke 4 remained colorless, demonstrating the specific expression of *GmSg-1*^*a*^ gene in these four soybean cultivars (Figure 3). Similarly, in the presence of probes of GAb1 and GAb2, the colorless to green change was observed in tubes 9, 10, 11, and 12, and tubes 7 and 8 containing Wuhei and Jinda 73 remained colorless, indicating the specific expression of *GmSg-1*^*b*^ gene in Jinyi 30, Jinke 4, WS 285, and Jinda 47 (Figure 3). The results of agarose gel analysis are consistent with those of colorimetric determination (Figures 2(c)–2(d)). For the cultivars Jinyi 30 and Jinke 4, no HCR products for the *GmSg-1*^*a*^ sequences were detected in the assay mixture containing GAa1 and GAa2, while HCR products of *GmSg-1*^*b*^ sequences were detected in the presence of GAb1 and GAb2. On the contrary, for the cultivars Wuhei and Jinda 73, HCR products of *GmSg-1*^*a*^G sequence instead of *GmSg-1*^*b*^ sequence were detected. For the cultivars WS 285 and Jinda 47, both *GmSg-1*^*a*^ sequence and *GmSg-1*^*b*^ sequence were detected. Generally speaking, if the tested sample contains Aa saponin, the color of HCR system will change from colorless to green in the presence of probes GAa1 and GAa2, while it will remain colorless after adding probes GAb1 and GAb2; however, the tested sample containing Ab saponin is the opposite. Therefore, hybridization chain reaction can be used to detect *GmSg-1*^*a*^ and *GmSg-1*^*b*^.

### 3.4. Visual-Chip-Based Assay of *GmSg-1* Genes

**Figure 4:**
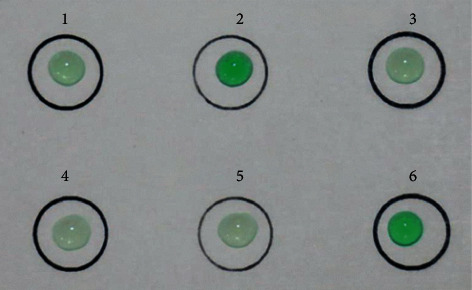
Visual-chip based detection of *GmSg-1*. Each circle was coated with a mixture of HCR systems containing different hairpin probes and target genes with different sequences. Circles 1 and 4: the probes in the system were replaced with sterile ultrapure water as blank; target genes were GAa1/GAa2 and GAb1/GAb2, respectively. Circles 2 and 3: the probes in the system were all GAa1/GAa2; the target genes were TAa and Tab, respectively. Circles 5 and 6: the probes in the system were all GAb1/GAb2; the target genes were TAa and Tab, respectively.

To facilitate the use of HCR system for detection, we tried to make a gene chip based on HCR system. The pretreated DNA probes GA1/GA2 and GAb1/GAb2, which were designed to detect the *GmSg-1*^*a*^ and *GmSg-1*^*b*^, mixed with 5 × Tris-HCl buffer and then printed on a plastic sheet in advance, and then the hybridization chain reaction would be generated by pipetting the target genes TAa and TAb. The colorimetric assay of HCR could be performed on the sheet after adding the corresponding reagents of oxidation reaction, as shown in Figure 4. The results are consistent with the specificity experiments; the circles which were printed GAa1/GAa2 and GAb1/GAb2 show green in the presence of corresponding genes TAa and TAb, respectively. However, when the circles printed GAa1/GAa2 and GAb1/GAb2 were added to opposite target genes TAb and TAa, the results are as colorless as the blank. Therefore, the HCR system can also be made into a gene chip for the detection of *GmSg-1*^*a*^ and *GmSg-1*^*b*^.

## 4. Conclusions

In this study, we studied the feasibility of HCR system to identify class A soybean saponins by detecting their corresponding encoded *GmSg-1* genes. It was found that hybridization chain reaction has excellent specificity in detecting genes, and the results were confirmed by agarose gel (3%) electrophoresis, so it could be used to identify the *GmSg-1* in different soybean cultivars. Furthermore, a convenient visual chip based on hybridization chain reaction was made by printing the probes designed to *GmSg-1* sequence on plastic sheet; the colorimetric assay could be implemented by the naked eye without resorting to expensive equipment, which has the advantages of high sensitivity, speed, and simplicity. In addition, the application of hybridization chain reaction on quantitative analysis of gene *GmSg-1* should be further studied.

## Figures and Tables

**Table 1:** Probes and target fragments required for HCR reactions.

Sequence name	*Gmsg-1* gene probe sequence (5′ to 3′)
TA92F	5′-ATCCCACGACTTGCCTTCA-3′
TA92R	5′-CCCGTGTCGGAATGGAGT-3′
GAa1	5′-CCTCTTCGCCGTCAGCGCCATGAAGTCCGTGGGTAGGGCGGGTTGGGAAATTACCCACTTCATGGCGCTGACGGCG-3′
GAa2	5′-CGGACTTCATGGCGCTGACGGCGAAGAGGCGCCGTCAGCGCCATGAAGTCGGTGGGTA-3′
TAa	5′-CGGACTTCATGGCGCTGACGGGGTGAGATATG-3′
TAa92	5′-CCCGTGTCGGAATGGAGTTCGGGGTGAGATATGACGGACTTCATGGCGCTGACGGCGAAGAGGGGGTATGAGTTGAAGGCAAGTCGTGGGAT-3′
GAb1	5′-CTCTTCTCCGGCGCCGCCATGAAGTGCGTGGGTAGGGCGGGTTGGGAAATTACCCACCCACTTCATGGCGGCGCCGGAG-3′
GAb2	5′-GCACTTCATGGCGGCGCCGGAGAAGAGG-3′
TAb	5′-GCACTTCATGGCGGCGCCGGAGAAGAGG-3′
TAb92	5′-CCCGTGTCGGAATGGAGTTCGGGGTGAGATATGACGCACTTCATGGCGGCGCCGGAGAAGAGGGGGTATGTTGAAGGCAAGTGGGAT-3′

## Data Availability

The data used to support the findings of this study are included within the article.
